# The effectiveness of pay-for-performance contracts with non-governmental organizations in Afghanistan – results of a controlled interrupted time series analysis

**DOI:** 10.1186/s12913-023-09099-y

**Published:** 2023-02-07

**Authors:** Diwa Samad, Bashir Hamid, Ghulam Dastagir Sayed, Yueming Liu, Wu Zeng, Alexander K. Rowe, Benjamin Loevinsohn

**Affiliations:** 1 The Global Fund to Fight AIDS, Tuberculosis and Malaria, Geneva, Switzerland; 2 Ministry of Public Health of Afghanistan, Kabul, Afghanistan; 3 The World Bank, Kinshasa, Democratic Republic of Congo; 4 Feinberg School of Medicine, Northwestern University- United States, Chicago, USA; 5 Department of Global Health, Georgetown University- United States, Washington, DC USA; 6 Gavi, The Vaccine Alliance, Geneva, Switzerland

**Keywords:** NGOs, Contracting, Health, Afghanistan, Pay-for-performance, Results-based financing, Fragile settings

## Abstract

**Background:**

In many contexts, including fragile settings like Afghanistan, the coverage of basic health services is low. To address these challenges there has been considerable interest in working with NGOs and examining the effect of financial incentives on service providers. The Government of Afghanistan has used contracting with NGOs for more than 15 years and in 2019 introduced pay-for-performance (P4P) into the contracts. This study examines the impact of P4P on health service delivery in Afghanistan.

**Methods:**

We conducted an interrupted time series (ITS) analysis with a non-randomized comparison group that employed segmented regression models and used independently verified health management information system (HMIS) data from 2015 to 2021. We compared 31 provinces with P4P contracts to 3 provinces where the Ministry of Public Health (MOPH) continued to deliver services without P4P. We used data from annual health facility surveys to assess the quality of care.

**Findings:**

Independent verification of the HMIS data found that consistency and accuracy was greater than 90% in the contracted provinces. The introduction of P4P increased the 10 P4P-compensated service delivery outcomes by a median of 22.1 percentage points (range 10.2 to 43.8) for the two-arm analysis and 19.9 percentage points (range: - 8.3 to 56.1) for the one-arm analysis. There was a small decrease in quality of care initially, but it was short-lived. We found few other unintended consequences.

**Interpretation:**

P4P contracts with NGOs led to a substantial improvement in service delivery at lower cost despite a very difficult security situation. The promising results from this large-scale experience warrant more extensive application of P4P contracts in other fragile settings or wherever coverage remains low.

**Supplementary Information:**

The online version contains supplementary material available at 10.1186/s12913-023-09099-y.

## Strengths and limitations of the study

- We have 48 months of data before introduction of P4P and 30 months of data afterwards. The HMIS data we use were verified robustly and independently.
- The scheme was implemented on a very large scale — covering a population of about 29 million in the intervention provinces and 1.4 million in the comparison arm. It was also implemented at a cost that is reproducible and was done in the context of increasing insecurity. This was not a lavish pilot.
- We rely primarily on HMIS data as no household survey data are available after 2018 to ascertain the population-level impact of the scheme
- The HMIS data from the comparison provinces were less consistent and accurate than those from the intervention provinces, which could bias the estimates (and understate the differences).
- There is some heterogeneity in the results between services.
- Non-availability of valid sub-population estimates for denominators

## Introduction

Fragile and conflict-affected settings, like Afghanistan, contribute disproportionately to worldwide poverty and the global burden of disease. In 2021, about 48% of the extreme poor lived in such settings, and this is expected to increase to 66% by 2030 [1]. Of the 10 countries with the highest rates of infant mortality, seven are classified as fragile states; and 14 of the 20 countries with the weakest progress on reducing maternal mortality from 1990 to 2015 were fragile [2]. Finding effective ways of improving health services in fragile settings is thus of global consequence.

Significant attention has recently focused on using financial incentives to improve the delivery and uptake of health services in fragile and low-resource settings. This has usually been referred to as results-based financing although the literature is replete with different nomenclature. In this paper, we examine pay-for-performance (P4P) contracts with non-governmental organizations (NGOs) in which they are paid partly on the basis of the number of defined services they deliver.

Much of the recent literature on results-based financing focuses on performance-based financing (PBF), in which funds are provided to individual public sector health facilities based mostly on the number of services they deliver. A recent systematic review of PBF [3, 4] combined the results of 22 randomized studies. The authors concluded that PBF had a modest effect on utilization. PBF increased institutional deliveries by only 4.4% points and uptake of modern contraceptives by 2.4% points. This is consistent with a recent Cochrane Collaboration systematic review [5] which found, based on a review of 14 randomized studies predominantly in the public sector, limited effects on institutional deliveries (− 3 to + 18%), modern family planning use (0.2%), and pentavalent vaccination coverage (− 5.7%). Numerous articles have highlighted the importance of context in assessing PBF impact [6–8].

We evaluated whether contracts with NGOs that use an approach similar to PBF would be effective. The P4P contracts were similar to PBF in that they made incentive payments based on the number of specifically selected services (such as institutional deliveries) that were provided to beneficiaries and the services had to be verified independently. The P4P contracts differed from PBF in that: (i) NGOs rather than the public sector provided the services; and (ii) payments were made to the NGO for a defined geography, in this case a whole province rather than to an individual health facility.

There has been interest in harnessing the non-state sector to improve health services, however, a recent systematic review [9] of contracting with NGOs found only two studies [10, 11] that used rigorous methodologies and concluded more studies were needed. The quasi-experimental study from Cambodia [10] randomized districts to contracts with NGOs or continued public sector management. Contracting led to an average 0.5 baseline standard deviation increase in coverage compared to the control districts (e.g., Vitamin A coverage increased by 21% points and antenatal care by 33% points). Similarly, a quasi-experimental study from Guatemala [11] found that mobile outreach teams implemented by contracted NGOs led to an increase in prenatal care provided by a nurse or physician (29% points), in women receiving 3 or more prenatal visits (38% points), and DTP3 coverage (24% points), but no discernable increase in the uptake of family planning.

NGOs have played an important role in health care in Afghanistan over the last 40 years. From the 1980s until the early 2000s, most health services in Afghanistan, about 80% in rural areas, were provided by international humanitarian NGOs that worked “cross-border,” primarily from Pakistan. Starting in 2002, with the establishment of a new government, the Ministry of Public Health (MOPH) defined its priorities through the Basic Package of Health Services (BPHS) and Essential package of Hospital services (EPHS) which laid out structures of the country’s primary health care and secondary hospital services [12]. With financial support from the international community, the Government contracted with NGOs for the delivery of these two packages of services. By setting priorities, allocating geographical responsibilities, providing financing, and carefully monitoring performance, the MOPH was able to provide strategic direction to what was previously an uncoordinated sector and helped address serious constraints, such as scarce human resources and a lack of physical facilities [13].

Health services and health outcomes in Afghanistan improved considerably from 2004 to 2015, although significant gaps remained [14]. For example, the under-five mortality rate as measured by demographic and health surveys declined from 97 per 1000 live births in 2010 to 55 in 2015, but the maternal mortality ratio remained high [15]. Starting in 2015, a project called SEHAT (“health” in Dari) pooled funds from different donors so that contracts were the same across the country. A presidential health summit in 2017 identified key areas where progress was lagging: 1) family planning to provide women with greater reproductive choice; 2) institutional deliveries to decrease maternal mortality; 3) growth monitoring to address widely prevalent child malnutrition; and 4) immunization. There was also consensus on the need to shift from “contract management” (adherence with contractual obligations) to “performance management” (greater focus on results). This shift provided the impetus for the design of a new health project called “SEHATMANDI” (“healthy” in Dari), which introduced an explicit P4P aspect to the NGO contracts. These contracts began on January 1, 2019 and covered about 29 million people in 31 of the country’s 34 provinces.

**Fig. 1:**
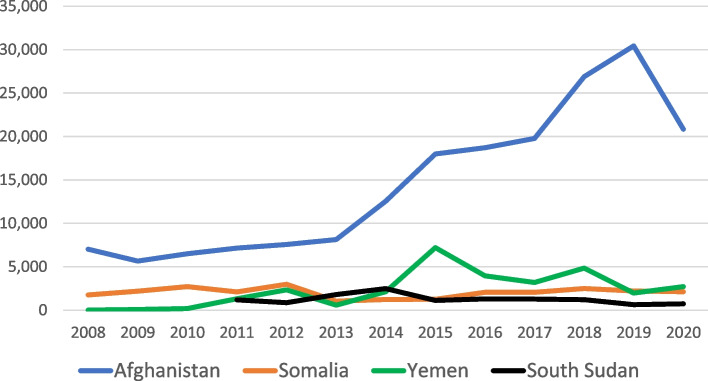
Number of Conflict-Related Fatalities per Year from the Uppsala Conflict Data Program^a^

P4P contracts were implemented during a period of increasing insecurity in Afghanistan. The Uppsala Conflict Data Program [16] found that total fatalities from conflict increased annually from 2008 to 2019 before declining in 2020 (Fig. 1). The conflict in Afghanistan was considerably bloodier than those in Somalia, South Sudan, and Yemen; and in 2018, Afghanistan replaced Syria as the world’s deadliest conflict.

We conducted an interrupted time series analysis with a non-randomized comparison group to assess the effectiveness of a P4P scheme with NGOs in Afghanistan in increasing the volume of services. We compared this approach to a “business as usual” model of government delivery of services. This contributes to the literature on the use of financial incentives and contracting with non-state actors for health service delivery.

## Methodology

### Interventions

#### P4P contracts under SEHATMANDI (2019–2021)

The P4P contracts in Afghanistan have been described in detail elsewhere [17]. Briefly, they involved paying the selected NGOs a fixed tariff for each of the 11 services, mostly focused on reproductive, maternal, neonatal, and child health. The number of services claimed by the NGO was verified by an independent third-party under a separate contract with the MOPH. Payments to the NGO were adjusted based on the findings of the third-party. For example, if an NGO claimed 1000 institutional deliveries in 6 months, the tariff per delivery was $20, and the third-party was able to verify 90% of the deliveries in the health facilities they visited, then the NGO would receive $18,000 (1000 x $20 × 90%). In addition to the P4P payments, NGOs were provided a lump-sum payment to cover their overheads and other services not compensated by the P4P mechanism. The amount of the lump sum was determined through a competitive tendering process and averaged 40.2% of the total contract value (range 4 to 69%).

#### SEHAT contracts (2015–2018)

SEHAT contracts differed from SEHATMANDI contracts principally in the way NGOs were paid. Under SEHAT, NGOs received 80% of the price that they bid as a lump-sum payment every 6 months. They could earn up to 10% of the contract value based on the functionality of their health facilities as judged by the availability of technical staff, functional equipment, and pharmaceuticals. They could earn a further 10% of the contract price based on reaching targets using HMIS data. This was not a PBF approach because it was based on achievement of pre-specified targets not a payment for each service provided. The approach resulted in less of the payments to the NGOs being at risk. In practice, contracted NGOs earned about 96% of the contract value. BPHS contracts under SEHAT cost 15% more than under SEHATMANDI.

#### MOPH-SM

The MOPH Strengthening Mechanism (MOPH-SM) involved providing a fixed annual budget and technical assistance to the provincial health directorates of 3 provinces (Kapisa, Panjshir, and Parwan) with a combined population of 1.4 million. These provinces were considered the comparison area in this study. They were chosen by the Government in 2004 as a testbed for public sector delivery of services and were maintained that way until the fall of the Government in 2021. The MOPH-SM provinces were: 1) close to Kabul; 2) relatively easy to access; and 3) suffered less insecurity than other provinces. The government continued to deliver health services and competitively recruited 25 Afghan consultants to support the provinces. The government paid the salaries of about two-thirds of the frontline health workers, while international donors paid for the technical assistance, drugs, supplies, salaries for about one- third of the frontline workers, and other operating expenses. The main differences between the three approaches are summarized in Table 1.

**Table 1:** Characteristics of the different types of contracts

Characteristic	SEHAT (2015–2018)	SEHATMANDI (Intervention) (2019–2021)	MOPH-SM (2015–2021) (Comparison)
Performance Aspect of Contract	Up to 10% of contract value based on functionality of health facilities. Up to 10% could be earned based on achievement of pre-set targets. NGOs received average of 80% of “performance” payment (96% of total contract value).	A fixed tariff for each verified service provided. Pay for Performance (P4P) accounted for an average of 60% of total contract value	No performance payment
Lump sum payment	Based on total bid price, represented at least 80% of total contract value.	Average of 40% of total contract value. Determined through competitive bidding.	Annual fixed budget
Who was contracted?	NGOs (71% of contracts awarded to Afghan NGOs, 17% to International NGOs, and 12% to joint ventures.)	NGOs (68% of contracts awarded to Afghan NGOs, 22% to international NGOs, and 10% to joint ventures.)	25 individual Afghan consultants to support 3 provinces.
Who did the contracting?	MOPH through a grants & contracts management unit (GCMU)	MOPH through a GCMU but performance was managed by a performance management office (PMO)	MOPH through an MOPH-SM unit
What was contracted for?	31 BPHS^a^contracts and 16 separate EPHS^b^ contracts signed with different NGOs	Combined BPHS^a^ and EPHS^b^ contracts in 15 provinces and 16 stand-alone BPHS^a^ contracts.	Combined BPHS^a^ & EPHS^b^
How were contractors selected?	Competitive “quality and cost-based selection” where bid price was 25% of the total score	Competitive “quality and cost-based selection” where the “lump sum” bid price was 20% of the total score.	Consultants were selected through quality-based selection
Autonomy provided to providers	Substantial autonomy around organization & procurement. Had to follow BPHS^a^ & EPHS^b^ staffing norms	Same as during SEHAT.	Limited, bound by civil service regulations
How was verification done?	Third-party monitor visited randomly selected health facilities to review records for consistency with HMIS reports. Randomly selected patients were visited at home to see whether they had received the services listed in the health facility records.		
Ownership of the health facilities	Ministry of Public Health (MOPH)	MOPH	MOPH
Geographic Extent	31 provinces	31 provinces	3 provinces
Cost of BPHS^a^ Contracts (Average)	US$5.28 per capita per year	US$4.48 per capita per year	Not applicable
Cost of combined BPHS^a^ & EPHS^b^ contracts (Average per capita per year)	Not applicable	US$7.12	US$7.26 (2019–2021)

^a^*BPHS* Basic Package of Health Services, a series of primary health care services delivered through health posts, sub-centers, basic health centers, comprehensive health centers, and district hospitals

^b^*EPHS* Essential Package of Hospital Services, a series of hospital-based services delivered through secondary provincial hospitals and regional hospitals

### Data sources

We analyzed quarterly HMIS data collected by the MOPH from January 1, 2015, to June 30, 2021, for both the intervention and comparison provinces. Each facility submitted standard HMIS forms through the contracted NGO to the provincial health units. From there, the forms were submitted electronically to the central MOPH. All P4P indicators (see Table 3, column 1) were included in the HMIS. One of the 11 indicators, growth monitoring and promotion, was introduced as a new indicator only in 2019 and so could not be analyzed. Prior to doing the analysis, we chose 4 non-compensated services (last 4 rows of Table 3) for which there was HMIS data during the study period to determine whether they were negatively affected.

Verification of the HMIS data was performed by an independent third-party contracted by the MOPH. Every 6 months, the third-party chose a stratified random sample of 25% of the health facilities in each province (with a minimum of 10 facilities) and visited them in person. For the 11 P4P indicators, the third-party compared the quantity of services found in the hard copy registers in the facility to the numbers reported through the HMIS. This ratio was defined as “consistency” and was capped at 100% (“consistency” above 100% would have represented under-reporting of performance which was seen as less serious than over-reporting.)

The third-party also randomly selected 5 patients for each of the 11 services (55 patients in total) and visited them at home to see whether they had received the specified service at the indicated time. The ratio of patients found to exist and to have received the service to the number of patients sampled was referred to as “accuracy.” A composite score of HMIS data “correctness” equaled the product of the consistency and accuracy scores. The verification procedures were similar during SEHAT and SEHATMANDI except that during SEHAT, only 20 patients were sampled to assess the accuracy of the facility records and only 3 randomly chosen indicators were assessed. The verification during both projects covered all provinces, including the comparison group, and the verification cost was about $600,000 per year, 0.5% of the annual total contract value ($120 million).

The consistency and accuracy of the HMIS data were above 95% in the contracted provinces but somewhat lower in the MOPH-SM provinces (Table 2) indicating more over-reporting. There is no evidence that the consistency, accuracy, or “correctness” of the HMIS data deteriorated with the introduction of P4P contracts.

**Table 2:** Verification of HMIS Data by an Independent Third-Party Monitor in Intervention (P4P) and Comparison (MOPH-SM) Provinces

Project/Years	Provinces	Median (inter-quartile range)		
		Consistency ^**a**^	Accuracy ^**b**^	Composite ^**c**^
SEHATMANDI 2019–2021	P4P (Intervention)	98.1% (94.4–99.3%)	99.1% (97.2–100%)	96.3% (91.9–99.0%)
	MOPH-SM (Comparison)	86.0% (84.5–89.9%)	95.7% (89.5–99.0%)	83.67% (76.6–86.6%)
SEHAT 2015–2018	Contracted to NGOs (pre-P4P)	91.5% (86.3–96.3%)	98.1% (94.3–100%)	88.7% (80.9–94.6%)
	MOPH-SM (Comparison)	84.9% (81.9–86.6%)	92.6% (84.4–95.4%)	75.8% (69.8–81.0%)

*Abbreviations*: *HMIS* Health and management information system, *MOPH-SM* Ministry of Public Health Strengthening Mechanism, *P4P* Pay for performance

^a^Consistency is the ratio of the number of services recorded in health facility registers to those reported in the HMIS

^b^Accuracy is the ratio of the number of services verified by household visits to the patients and the number of patients sampled

^c^Composite score was the product of the consistency and the accuracy scores, which gives an overall measure of the “correctness” of the HMIS data

To assess quality of care, we used information from a series of annual health facility surveys that were known as the Balanced Scorecard. The facility surveys were conducted by the third-party in PHC facilities (to assess BPHS implementation) and separately in hospitals (to assess EPHS execution). They involved direct observation of patient-provider interactions, examination of the facility, exit interviews with patients, and interviews with staff and community members.

For the BPHS Balanced Scorecard, the third-party visited an average of 24 facilities per province (about 30% of all PHC facilities) and examined 23 composite indicators. For EPHS, the third-party visited all provincial hospitals and regional hospitals (in those provinces where they were located) and examined 34 composited indicators. Even at the height of the conflict (in 2019 and 2020) the third-party was able to visit 99.2% of the sampled facilities. The cost of carrying out the Balanced Scorecard was about $400,000 per round.

### Analysis

We analyzed quarterly HMIS data points as rates (e.g., number of institutional deliveries/100,000 population) with segmented linear regression modelling. The rates were calculated based on the population size for the corresponding year when services were delivered. The baseline time segment was January 2015–December 2018, scale-up was January–March 2019 (excluded from the analysis), and follow-up was April 2019–June 2021. Models were first run with a term to adjust for autocorrelation from repeated observations over time; but if the autocorrelation term was not significant at the *p* < 0.05 level, it was dropped. The modeling was performed separately for each study arm, and the results were used to estimate three effect sizes for each outcome: 1) level change from the end of the baseline period to immediately after scale-up (Table 3, column 2), 2) baseline-to-follow-up change in outcome slope (Table 3, column 3), and 3) a relative-change “combined” effect size of the level and slope changes at the mid-point of the follow-up period (Table 3, column 4). A fourth effect size was the arithmetic difference between the “combined” effect sizes of the intervention and comparison arms (Table 3, column 5). 95% confidence intervals were calculated with standard errors that were estimated using the delta method, which estimates the variance of a function by combining the uncertainty of each element of the function [18]. We computed the combined effect size at 4.5 quarters because that was the mid-point of the follow-up period. This approach was used because other studies [19, 20] have found that effect sizes of ITS studies calculated at the mid-point of the follow-up period are similar to effect sizes derived from a randomized-controlled study design. The overall analytic approach is identical to that used by a large systematic review of the effectiveness of strategies to improve health worker performance in LMICs [21].

**Table 3:** Modeling results on the effects of the Pay for Performance (P4P) intervention – Services provided per 100,000 population

Outcome indicator (1)	Analysis of the intervention arm only			Analysis of the intervention and comparison arms	R^2^ for intervention arm (6)	R^2^ for comparison arm (7)
	Level effect (95% CI) (2)	Slope effect (95% CI) (3)	Combined effect (95% CI) (4)	Combined effect (95% CI) (5)		
Couple Years of Protection	119.6 (88.9 to 150.3)	3.3 (−1.5 to 8.1)	56.1% points (41.4 to 70.9)	43.8% points (22.3 to 65.4)	0.953	0.371
Cesarean Section	0.4 (−1.9 to 2.6)	0.9 (0.6 to 1.3)	30.0% points (14.5 to 44.6)	27.0% points (0.0 to 594.8)	0.969	0.823
Antenatal Care total visits	409.6 (225.5 to 593.6)	37.7 (8.9 to 66.5)	30.0% points (20.3 to 40.0)	28.9% points (14.7 to 43.2)	0.905	0.506
Postnatal Care	222.1 (144.9 to 299.3)	18.0 (5.9 to 30.0)	26.9% points (20.0 to 33.7)	26.5% points (15.8 to 37.2)	0.957	0.789
Institutional deliveries	60.6 (30.9 to 90.2)	8.7 (4.0 to 13.3)	20.2%-points (14.4 to 26.0)	12.1%-points (0.9 to 23.2)	0.965	0.777
Tetanus Toxoid 2+ doses	263.3 (−10.1 to 536.6)	57.0 (14.3 to 99.7)	19.6% points (9.7 to 29.5)	14.1% points (−5.4 to 33.6)	0.659	0.04
TB Treated Cases	2.0 (0.5 to 3.5)	−0.2(−0.4 to 0.1)	10.7% points (0.0 to 21.9)	11.1% points (−17.3 to 39.6)	0.795	0.550
Under 5 Outpatient visits	1026.8 (−40.7 to 2094.3)	25.8 (− 141.0 to 192.6)	10.3% points (1.4 to 19.2)	21.4% points (6.3 to 36.5)	0.787	0.303
Pentavalent 3 immunization	5.6 (−55.7 to 67.1)	11.7 (2.1 to 21.3)	6.2% points (0.4 to 12.1)	10.2% points (1.2 to 19.3)	0.377	0.559
Major surgery	−4.8 (−12.9 to 3.4)	0.1 (−1.3 to 1.3)	−8.3% points (−20.0 to 3.4)	22.8% points (2.3 to 43.3)	0.768	0.574
**Non-Compensated Services**						
Measles vaccination for. Children < 12 months	−87.0 (− 178.9to 4.9)	12.9 (−1.5 to 27.2)	−3.3% points (− 12.1 to 5.5)	0.0% points (− 12.6 to 13.6)	0.442	0.288
New patients/clients	− 456.0 (− 3129.2 to 2217.1)	− 642.5 (− 1060.3 to − 224.7)	−9.2% points (− 15.2 to − 3.2)	− 0.2% points (− 13.9 to 13.5)	0.440	0.114
Minor surgeries	− 73.1 (− 118.3 to − 27.9)	− 12.5 (− 19.6 to − 5.4)	− 51.1% points (− 62.2 to − 39.9)	−74.8% points (− 126.5 to − 23.1)	0.714	0.810
IPD – admissions	−24.5 (− 97.3 to 48.3)	2.3 (−9.1 to 13.6)	−2.5% points (− 13.2 to 8.3)	3.3% points (− 13.0 to 19.6)	0.512	0.162

Further details on the analytical approach have been described elsewhere [see Appendix 1, pages 52–56 and 69–72 of reference 21] and are in Table 4. A pre-planned sensitivity analysis was performed in which one data point (April–June 2020) was omitted because the COVID-19 pandemic had a particularly strong negative effect on utilization of health services during this quarter. We assessed the goodness-of-fit of our models using R^2^ statistics. Statistical analyses were conducted using R Statistical Software (version 4.1.2; R Foundation, Vienna, Austria).

**Table 4:** Details on the estimation of effect sizes from the main analysis of the institutional deliveries outcome

**Fig. 2:**
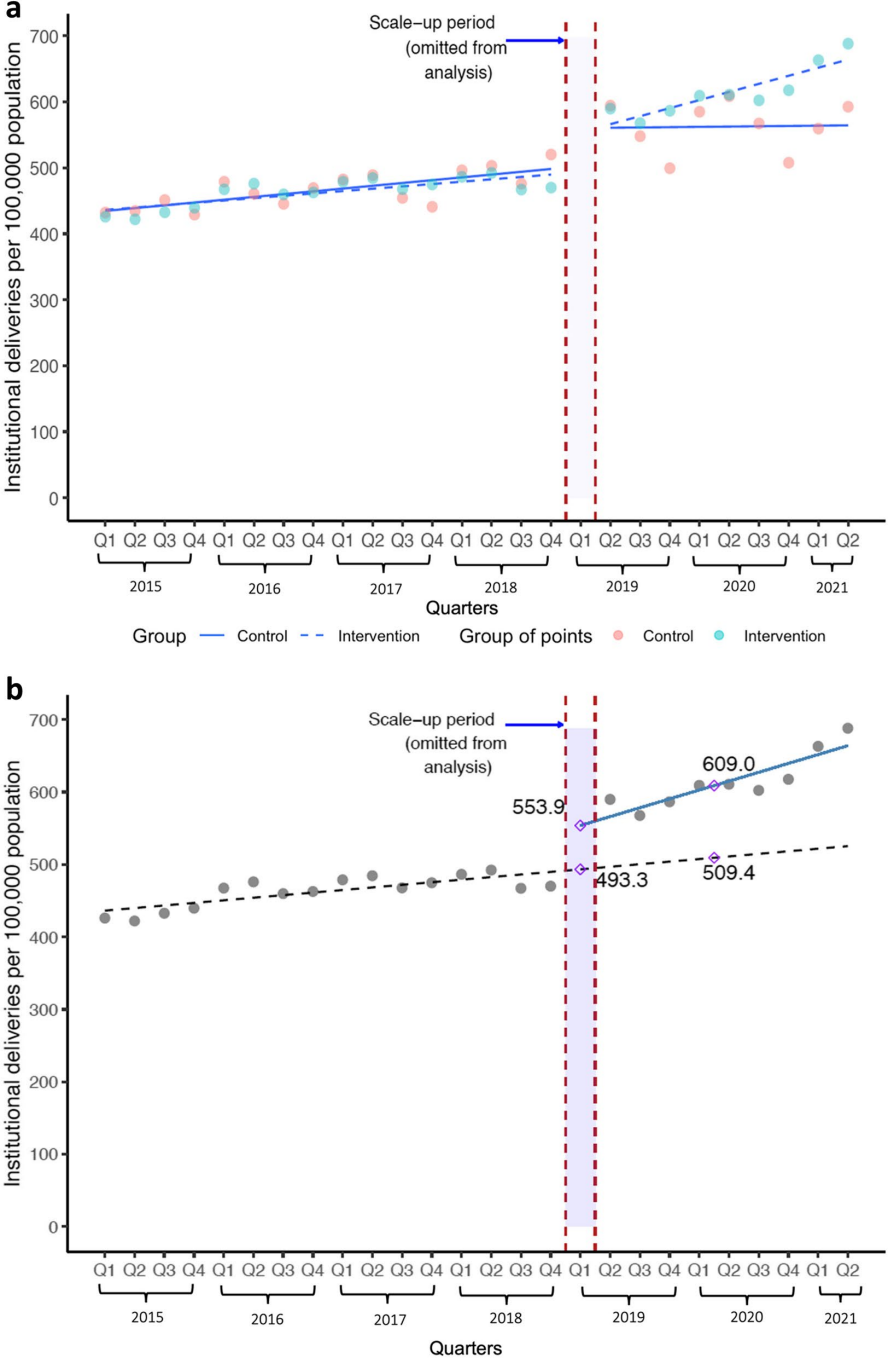
**a** Institutional deliveries in Contracted (Intervention) and MOPH-SM (Control) Provinces. **b** Institutional deliveries - Intervention(P4P) arm only

The segmented regression model is: Y = β_0_ + (β_1_ x G) + (β_2_ x time) + (β_3_ x G x time) + ε. Y = outcome; time = time in quarters, with the intervention starting at time = 0; G is an indicator variable with a value of 0 (during baseline, when time < 0) or 1 (after intervention scale-up, when time ≥ 0), and ε is an error term. β_0_ = predicted outcome value just before intervention scale-up, β_1_ = level effect size (Table 3, column 2), β_2_ = outcome slope during baseline, and β_3_ = slope effect size (Table 3, column 3). For institutional deliveries (main analysis), the model parameter estimates are: β_0_ = 493.3, β_1_ = 60.6, β_2_ = 3.6, and β_3_ = 8.7. Model-based estimates of the outcome are shown as diamonds at several key time points in Fig. 2a. • The level effect (60.6 deliveries per 100,000 population) is the difference in the outcome immediately after intervention scale-up (553.9) minus the outcome at the very end of the baseline period (493.3). • The slope effect (8.7 deliveries per 100,000 population per quarter) is the difference in the slope after intervention scale-up (β_2_ + β_3_, or 12.3) minus the baseline slope (β_2_, or 3.6). • The “combined” effect size for the intervention arm (Table 3, column 4) is the arithmetic difference between: a) the relative change in the outcome from the end of baseline (493.3 deliveries per 100,000 population) to the mid-point of the follow-up period (time = 4.5 quarters) based on modeled estimates of observed outcome values (609.0), which is a 23.5% increase—i.e., (609.0–493.3)/493.3; and b) the relative change in the outcome from the end of baseline to the follow-up mid-point based on the counterfactual, which is the baseline slope extended into the follow-up period (509.4), which is a 3.3% increase—i.e., (509.4–493.3)/492.3. Thus, the combined effect size is 23.5–3.3%, or a 20.2 percentage-point difference. • The 2-armed combined effect size (Table 3, column 5) is the combined effect size for the intervention arm (20.2 percentage points) minus the combined effect size for the comparison arm (8.1 percentage points [not shown in Table 3]), or 12.1 percentage points.	

No ethical approval of this study was sought because it relies on published HMIS data that had no individual identifiers and no disaggregation below the level of a province.

## Results

Figure 2a, shows a comparison in the number of institutional deliveries per 100,000 population in the contracted provinces and the MOPH-SM provinces. The levels and rates of change appeared similar during the SEHAT project (2015–2018). These rates of increase diverge sharply after the introduction of P4P in the first quarter of 2019 and the rate of change is accompanied by a substantial “step change.”

Figure 2b shows the level effect and slope effect in just the contracted provinces (the intervention arm). The implementation of P4P immediately increased the number of institutional deliveries by 60.6 deliveries per 100,000 population (95% confidence interval 30.9 to 90.2). There was also a change in the slope which increased 8.7 (95% CI: 4.0 to 13.3) deliveries per 100,000 population per quarter. Combining the level effect and slope effect, we estimate that the number of institutional deliveries was 609.0 per 100,000 population at the midpoint of P4P implementation (after 4.5 quarters) compared to 509.0 per 100,000 in the counterfactual scenario. The increase is equivalent to 20.2 percentage points (95% CI: 14.4–26.0%). Effect sizes are further described in Table 4 (with institutional deliveries as an example) and graphs for the other nine P4P indicators are in Additional file 2: Annex B.

Table 3 shows the impact of P4P on the 10 compensated services (including institutional deliveries as described above) and four non-compensated health services. Overall, the introduction of P4P was associated with median increase in service delivery of 22.1% points (range: 10.2 to 43.8%) in the two-arm analysis (Table 3, column 5). The one arm analysis of the P4P services showed a 19.9 percentage point (range: − 8.3 to 56.1) median increase in service delivery (Table 3, column 4). The greatest increase was in couple years of protection with an increase of 43.8% points (95% CI: 22.3–65.4%), and the smallest in pentavalent 3 immunization with an increase of 10.2% points (95% CI: 1.2–19.3%,). We examined services that were not compensated using P4P under SEHATMANDI (bottom of Table 3) and found that for three of the four indicators examined there was no statistically significant impact of P4P on the volume of services provided. Only in minor surgery was there a sharp reduction.

The sensitivity analysis showed that the COVID-19 pandemic had little impact on P4P effectiveness (See Additional file 1: Annex A). The services that the Government of Afghanistan particularly targeted for improvement, namely family planning (couple years of protection), institutional delivery, and Pentavalent 3 child vaccination, saw substantial improvements compared to the comparison provinces of 44, 12, and 10 percentage points, respectively.

**Fig. 3:**
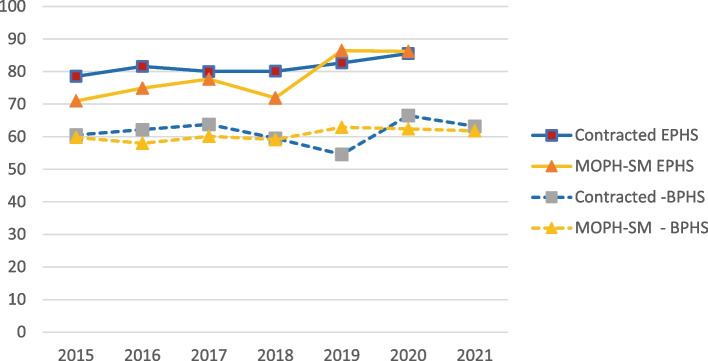
Mean Balanced Scorecard Scores (out of 100)^a^ for BPHS & EPHS in Contracted (Intervention) and MOPH-SM (Comparison) Provinces

Results of the annual health facility surveys (Balanced Scorecard) show a decline in the quality of care in 2019 in BPHS facilities in P4P provinces (Fig. 3). However: (i) this decline began in 2018 (before the P4P contracts began); (ii) the decline was short-lived; and (iii) the BSC scores rose after the first year of the contracts. We do not observe a decline in EPHS quality.

## Discussion

Our analysis indicates that P4P contracting with NGOs in Afghanistan was very effective in increasing the availability of key services, particularly those related to reproductive, maternal, and child health. In the medium term, the progress in increasing the quantity of health services impaired neither the quality of care nor progress on other health services. The improvements from baseline for the priority services were significantly larger in the P4P (intervention) provinces than those in the MOPH-SM (comparison) provinces despite lower per capita investments. The P4P results in Afghanistan are impressive given the deteriorating security situation during much of the study period and 15% lower cost per capita of the P4P contracts.

Our study has several strengths: (i) we have 48 months of data before P4P and 30 months of data afterwards; (ii) the HMIS data were verified robustly and independently; (iii) the intervention was done on a very large scale — covering a population of about 29 million in the P4P arm and 1.4 million in the MOPH-SM arm; (iv) the goodness of fit of the models we employed was generally good; (v) we are conservative in calculating effect sizes as we estimate impact only at the mid-point of implementation (not at the end); and (vi) besides the introduction of P4P, there were few other important changes between SEHAT and SEHATMANDI.

Our conclusions are tempered by limitations in our data, which include: (i) we rely primarily on HMIS data as no household survey data are available after 2018 to ascertain the population-level impact of P4P; (ii) the HMIS data from the MOPH-SM provinces were less consistent and accurate (thus, more likely to be overstated) than those from the contracted provinces, which could bias the estimates (and understate the differences); (iii) we do not have estimates of sub-populations (such as infants or pregnant women) with which to construct the denominators for coverage estimates and (iv) there is some heterogeneity in the results (see below).

The median effect sizes we found for P4P contracts with NGOs (22.1 and 19.9 percentage points), are consistent with previous quasi-experimental studies on the impact of contracting with non-state actors [10, 11]. They are larger than those found in the systematic reviews of PBF. We hypothesize that the differences between our results and the findings in the PBF literature are due to: (i) a greater ability of NGOs to take advantage of a P4P scheme possibly because of greater flexibility and autonomy (for example, in managing their human resources) and stronger management (for example, some NGOs extended opening hours or invested in real-time data solutions); and (ii) more of their payment was at risk (on average 60% was linked to performance) than is the case in most PBF schemes (where health worker salaries typically increase between 10 and 30% and there is no risk of earning less than they did before). It appears that P4P worked as a strong and effective signal to the providers about what was important and could explain the substantial “step change” in the first quarter of 2019 after the introduction of P4P.

The improvements that we observed are also large compared to other possible interventions. For example, the removal of user fees has been estimated to increase service delivery by about 15 percentage points [22]. The effect sizes we observed were achieved in a context where publicly financed services were already free. The MOPH abolished user fees for primary health care in 2007, and the verification studies found no evidence of “under-the-table” payments.

Besides increasing key service delivery outcomes, we believe that P4P contracts strengthened the government’s stewardship of the health sector by ensuring all service providers remained focused on its priorities. We also observed that a strong focus on performance helped the government increase the alignment of its development partners with the government’s health sector strategy.

We observed heterogeneity in the response of different indicators to P4P incentives. For example, immunization (Penta3, measles, and tetanus toxoid) saw smaller improvements than other indicators. Penta3 coverage in the 2018 Afghanistan Health Survey [23] was 60.8% indicating that there was substantial room for improvement. We hypothesize that the relatively modest progress in immunization could be due to: (i) too low a tariff for vaccination relative to the effort required to reach the unimmunized; (ii) demand-side issues (such as lack of parental interest); (iii) there were many organizations providing vaccination services besides the contracted NGOs; and (iv) explicit Taliban prevention of outreach immunization efforts.

With all financial incentives, like P4P, there is a legitimate concern about unintended negative consequences. We did see an initial drop in quality of care. However, this was quickly rectified. Non-compensated services were not much affected by P4P, except minor surgeries. We are unsure about the reason for this decline, but it may be due to insufficient attention to uncompensated services during semi-annual reviews by the MOPH. This highlights the importance of regular review of contractor performance on all indicators. We also observed a 30 percentage-point increase in caesarean sections, which raises the specter of excessive reliance on surgical deliveries. The 2018 survey [23] found a C-section rate of 6.6%, suggesting that there was still some benefit to increasing access to this service. One commonly expressed concern with P4P is that the cost of verification is high and takes funds away from service delivery. We found that verification costs under SEHATMANDI were about 0.5% of the contract value.

The success of P4P contracts with NGOs in Afghanistan justifies their continuation, especially as they are widely accepted by most stakeholders. The results also warrant their extension to other fragile and conflict-affected settings. The compelling results achieved in Afghanistan, at scale, at reasonable cost, and despite serious security challenges, suggests that P4P contracts with NGOs could be considered wherever the coverage of basic health services remains a challenge.

### Research in context panel

#### Evidence before this study

Fragile and conflict-affected countries account for a large proportion of global poverty and burden of disease. Despite their importance to the achievement of global goals, there are few rigorous evaluations of approaches to improve service delivery in such contexts. In these, and other settings where the coverage of health services is low, there is a need to understand what works. There is growing interest and use of NGOs to provide health services, especially in fragile environments; but a recent systematic review found only two robust evaluations of such efforts. The evidence on pay-for-performance (P4P) is mixed, and a recent meta-analysis of P4P schemes in the public sector in lower and lower-middle income countries (LMICs) has shown that its impact on service coverage is only a few percentage points.

#### Added value of this study

We describe here a formal interrupted time series (ITS) analysis to assess the effectiveness of P4P contracts with NGOs in Afghanistan to improve service delivery. It benefits from independent verification of the routine information, more than 6 years of data, consideration of quality of care, implementation on a very large scale (covering almost 30 million people), and comparison to a set of provinces where P4P contracts were not implemented. The introduction of P4P increased the delivery of 10 key services by a median of 22.1 percentage points.

#### Implications of all the available evidence

P4P contracts with NGOs had a large impact (with some heterogeneity of effects) on service delivery in Afghanistan and are worthy of expansion in other fragile settings or contexts where coverage is low*.* We found it possible to employ an ITS design to assess the impact of large-scale policy reform in a fragile context and the cost of evaluation (verification) was modest. The discussion about the value of P4P approaches, such as performance-based financing (PBF), needs to consider whether it is being implemented by the public sector or by NGOs (or other non-state actors). The latter seems better able to take advantage of the P4P approach. The lessons from the extensive experience in Afghanistan emphasize the importance of: 1) being clear about priorities; 2) providing NGOs with substantial autonomy so they can respond flexibly to the context they’re working in; 3) managing contractor performance rather than just contractual obligations; and 4) investing the relatively modest funds needed for independent and robust verification and measurement of performance.

## Supplementary Information

**Additional file 1: Annex A.** Modeling results on the effects of the Pay for Performance (P4P) intervention – Services provided per 100,000 population with sensitivity analysis to examine the effects of COVID-19.

**Additional file 2: Annex B.** Graphs for other nine P4P indicators.

**Additional file 3.** Quarterly HMIS data 2015–2021.

## Data Availability

All data used during the current study are available and is uploaded as supplementary material “Quarterly HMIS data 2015-2021”.
