# Supplementary material for: The effectiveness of pay-for-performance contracts with non-governmental organizations in Afghanistan – results of a controlled interrupted time series analysis
Source: BMC Health Serv Res. 2023 Feb 7;23:122. doi: 10.1186/s12913-023-09099-y (PMC9902816; doi:10.1186/s12913-023-09099-y)
Supplement: Supplementary file 1 — Additional file 1: Annex A. Modeling results on the effects of the Pay for Performance (P4P) intervention – Services provided per 100,000 population with sensitivity analysis to examine the effects of COVID-19. [file 12913_2023_9099_MOESM1_ESM.docx]

**Annex A: Modeling results on the effects of the Pay for Performance (P4P) intervention – Services provided per 100,000 population with sensitivity analysis to examine the effects of COVID-19**

| Outcome  (1) | Main analysis | | | | Sensitivity analysis that removes the one time point when COVID-19 had a particularly strong effect on the utilization of health services | | | | R^2^ for Main analysis only | | |
| --- | --- | --- | --- | --- | --- | --- | --- | --- | --- | --- | --- |
|  | Analysis of the intervention arm only | | | Analysis of the intervention and comparison arms | Analysis of the intervention arm only | | | Analysis of the intervention and comparison arms | R^2^ for intervention arm  (10) | R^2^ for comparison arm  (11) | |
|  | Level effect  (95% CI)  (2) | Slope effect (95% CI)  (3) | Combined effect  (95% CI)  (4) | Combined effect  (95% CI)  (5) | Level effect (95% CI)  (6) | Slope effect (95% CI)  (7) | Combined effect  (95% CI)  (8) | Combined effect  (95% CI)  (9) |  |  |  |
| Institutional deliveries | 60.6  (30.9 to 90.2) | 8.7  (4.0 to 13.3) | 20.2 %-points  (14.4 to 26.0) | 12.1 %-points  (0.9 to 23.2) | 61.1 (30.4 to 91.7) | 8.7(3.9 to 13.4) | 20.3 %-points (14.3 to 26.3) | 13.3 %-points (2.5 to 24.2) | 0.965 | 0.777 | |
| Antenatal Care total visits | 409.6  (225.5 to 593.6) | 37.7  (8.9 to 66.5) | 30.0% points  (20.3 to 40.0) | 28.9% points  (14.7 to 43.2) | 443.8 (297.5 to 590.1) | 37.7  (15.0 to 60.4) | 31.8% points (24.0 to 39.5) | 28.2 points  (17.3 to 39.1) | 0.905 | 0.506 | |
| Couple Years of Protection | 119.6  (88.9 to 150.3) | 3.3  (-1.5 to 8.1) | 56.1% points  (41.4 to 70.9) | 43.8% points  (22.3 to 65.4) | 126.0 (103.7 to 148.3) | 3.2  (-0.2 to 6.8) | 58.8% points (48.0 to 69.6) | 43.8% points (26.2 to 61.4) | 0.953 | 0.371 | |
| Cesarean Section | 0.4  (-1.9 to 2.6) | 0.9  (0.6 to 1.3) | 30.0% points  (14.5 to 44.6) | 27.0% points  (0.0 to 548.4) | 0.7  (-1.3 to 2.7) | 0.9  (0.6 to 1.2) | 31.7% points (18.1 to 45.3) | 31.6% points (5.1 to 58.0) | 0.969 | 0.823 | |
| Under 5 Outpatient visits | 1,026.8  (-40.7 to 2,094.3) | 25.8  (-141.0 to 192.6) | 10.3% points  (1.4 to 19.2) | 21.4% points  (6.3 to 36.5) | 1,274.2 (609.6 to 1,938.8) | 25.8  (-77.2.0 to 128.8) | 12.6% points (7.0 to 18.1) | 21.3% points (9.1 to 33.6) | 0.787 | 0.303 | |
| Major surgery | -4.8  (-12.9 to 3.4) | 0.1  (-1.3 to 1.3) | -8.3% points  (-20.0 to 3.4) | 22.8% points  (2.3 to 43.3) | -3.1  (-9.0 to 2.8) | 0.0  (-0.9 to 0.9) | -5.3% points  (-14.0 to 3.3) | 23.9% points (4.8 to 43.0) | 0.768 | 0.574 | |
| Pentavalent 3 immunization | 5.6  (-55.7 to 67.1) | 11.7  (2.1 to 21.3) | 6.2% points  (0.4 to 12.1) | 10.2% points  (1.2 to 19.3) | 14.2  (-41.7 to 70.1) | 11.7  (3.0 to 20.4) | 7.1% points  (1.8 to 12.5) | 10.1% points  (1.7 to 18.6) | 0.377 | 0.559 | |
| Postnatal Care | 222.1  (144.9 to 299.3) | 18.0  (5.9 to 30.0) | 26.9% points  (20.0 to 33.7) | 26.5% points  (15.8 to 37.2) | 229.7 (154.5 to 304.9) | 18.0  (6.3 to 29.4) | 27.5% points (20.9 to 34.2) | 25.9% points (15.8 to 36.0) | 0.957 | 0.789 | |
| TB Treated Cases | 2.0 (0.5 to 3.5) | -0.2(-0.4 to 0.1) | 10.7% points  (0.0 to 21.9) | 11.1% points  (-17.3 to 39.6) | 2.1 (0.5 to 3.6) | -0.2  (-0.4 to 0.1) | 11.1% points (0.0 to 22.6) | 9.2% points  (-19.6 to 38.1) | 0.795 | | 0.550 |
| Tetanus Toxoid 2+ doses | 263.3  (-10.1 to 536.6) | 57.0  (14.3 to 99.7) | 19.6% points  (9.7 to 29.5) | 14.1% points  (-5.4 to 33.6) | 305.6  (66.2 to 545.0) | 57.0  (19.9 to 94.1) | 21.2% points (12.4 to 30.0) | 14.3% points (-4.7 to 33.3) | 0.659 | | 0.04 |
| Uncompensated (Non-P4P) Services | | | | | | | | |  | | |
| Measles vaccination for. Children < 12 months | -87.0  (-178.9to 4.9) | 12.9  (-1.5 to 27.2) | -3.3% points  (-12.1 to 5.5) | 0.0 % points  (-12.6 to 13.6) | -79.6  (-171.1 to 12.0) | 12.9  (-1.3 to 27.0) | -2.4% points (-11.2 to 6.3) | 0.5% points  (-12.6 to 13.6) | 0.442 | | 0.288 |
| New patients/clients | -456.0  (-3,129.2 to 2,217.1) | -642.5  (-1,060.3 to  -224.7) | -9.2% points  (-15.2 to -3.2) | -0.2% points  (-13.9 to 13.5) | 143.1  (-1,613.8 to 1,900.1) | -642.5  (-914.7 to -370.3) | -7.5% points (-11.5 to -3.6) | -0.5% points (-12.7 to 11.7) | 0.440 | | 0.114 |
| Minor surgeries | -73.1  (-118.3 to -27.9) | -12.5  (-19.6 to -5.4) | -51.1% points  (-62.2 to -39.9) | -74.8% points  (-126.5 to -23.1) | -69.2  (-114.0 to -24.4) | -12.5  (-19.4 to -5.5) | -49.5% points  (-60.8 to -38.3 | -78.7% points (-129.9 To -27.5) | 0.714 | | 0.810 |
| IPD -– admissions | -24.5  (-97.3 to 48.3) | 2.3  (-9.1 to 13.6) | -2.5% points  (-13.2 to 8.3) | 3.3% points  (-13.0 to 19.6) | -3.6  (-148.6 to 141.3) | 11.0  (-11.7 to 33.6) | 4.2% points  (-7.6 to 16.1) | 8.4% points  (-8.4 to 25.3) | 0.512 | | 0.162 |
