# Supplementary material for: The effectiveness of pay-for-performance contracts with non-governmental organizations in Afghanistan – results of a controlled interrupted time series analysis
Source: BMC Health Serv Res. 2023 Feb 7;23:122. doi: 10.1186/s12913-023-09099-y (PMC9902816; doi:10.1186/s12913-023-09099-y)
Supplement: Supplementary file 2 — Additional file 2: Annex B. Graphs for other nine P4P indicators. [file 12913_2023_9099_MOESM2_ESM.docx]

#
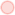


**Annex B:** Graphs for other nine P4P indicators

Couple Years of Protection

#
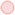


#
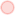


500


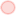


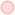

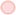


400


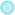


300

200


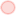

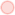

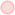

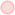

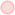

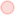

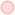

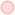

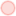

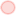

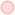

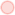

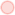

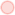

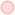

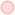

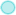

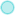

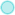

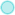

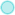

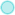

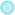

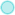

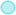

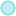

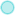

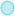

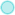

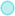

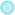

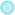

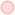

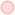

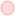

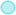

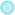

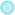

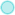

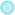

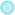

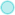

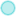


100

0

# Q1 Q2 Q3 Q4 Q1 Q2 Q3 Q4 Q1 Q2 Q3 Q4 Q1 Q2 Q3 Q4 Q1 Q2 Q3 Q4 Q1 Q2 Q3 Q4 Q1 Q2

Scale−up period (omitted from

analysis)


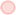

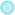
Group Control Intervention Group of points Control Intervention

Scale−up period (omitted from

analysis)

Cesarean Section

40

35


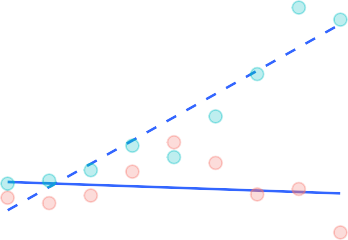
30

25


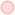
20


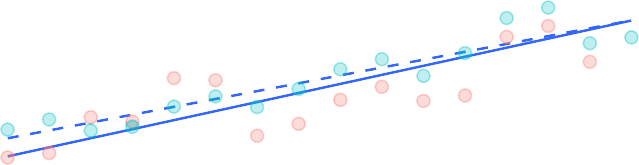
15

10

5

0

# Q1 Q2 Q3 Q4 Q1 Q2 Q3 Q4 Q1 Q2 Q3 Q4 Q1 Q2 Q3 Q4 Q1 Q2 Q3 Q4 Q1 Q2 Q3 Q4 Q1 Q2


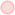

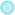
Group Control Intervention Group of points Control Intervention

Any ANC visits


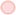


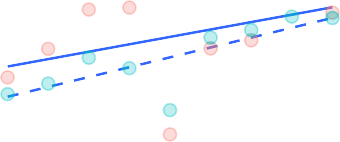
3000

Scale−up period (omitted from

analysis)


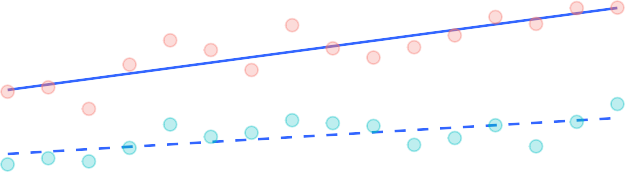
2500

2000

1500

1000

500

0

# Q1 Q2 Q3 Q4 Q1 Q2 Q3 Q4 Q1 Q2 Q3 Q4 Q1 Q2 Q3 Q4 Q1 Q2 Q3 Q4 Q1 Q2 Q3 Q4 Q1 Q2


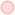

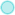
Group Control Intervention Group of points Control Intervention


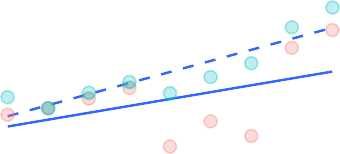
1800

Scale−up period (omitted from

analysis)

Postnatal Care

1600


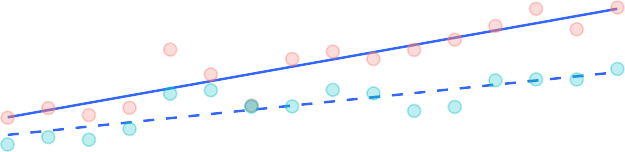
1400

1200

1000

800

600

400

200

0

# Q1 Q2 Q3 Q4 Q1 Q2 Q3 Q4 Q1 Q2 Q3 Q4 Q1 Q2 Q3 Q4 Q1 Q2 Q3 Q4 Q1 Q2 Q3 Q4 Q1 Q2


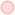

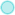
Group Control Intervention Group of points Control Intervention

Tetanus Toxoid 2+ doses


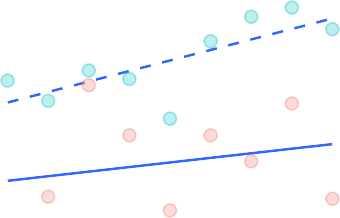
3500

Scale−up period (omitted from

analysis)


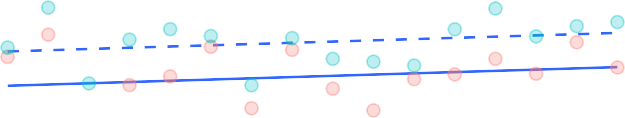
3000

2500


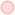


2000


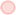


1500

1000

500

0

# Q1 Q2 Q3 Q4 Q1 Q2 Q3 Q4 Q1 Q2 Q3 Q4 Q1 Q2 Q3 Q4 Q1 Q2 Q3 Q4 Q1 Q2 Q3 Q4 Q1 Q2


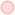

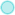
Group Control Intervention Group of points Control Intervention

Scale−up period (omitted from

analysis)

TB Treated Cases

30

25


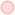


20


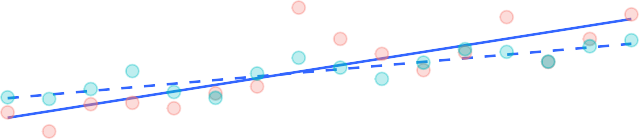

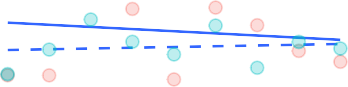
15

10

5

0

# Q1 Q2 Q3 Q4 Q1 Q2 Q3 Q4 Q1 Q2 Q3 Q4 Q1 Q2 Q3 Q4 Q1 Q2 Q3 Q4 Q1 Q2 Q3 Q4 Q1 Q2


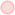

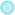
Group Control Intervention Group of points Control Intervention

Scale−up period (omitted from

analysis)

Under five visits


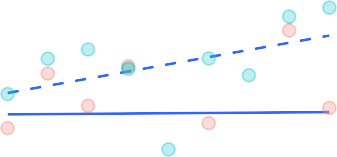
14000


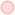


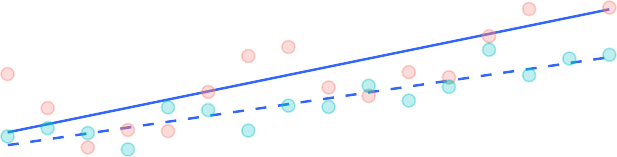
12000


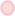


10000


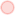
8000

6000

4000

2000

0

# Q1 Q2 Q3 Q4 Q1 Q2 Q3 Q4 Q1 Q2 Q3 Q4 Q1 Q2 Q3 Q4 Q1 Q2 Q3 Q4 Q1 Q2 Q3 Q4 Q1 Q2


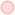

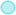
Group Control Intervention Group of points Control Intervention


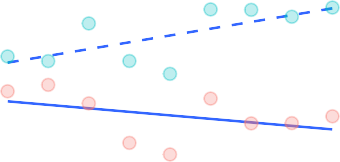
1100

Scale−up period (omitted from

analysis)

Pentavalent 3 immunization


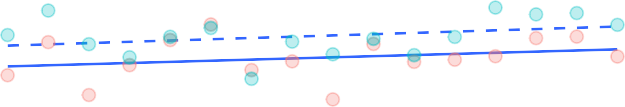
1000

900

800

700

600

500

400

300

200

100

0

# Q1 Q2 Q3 Q4 Q1 Q2 Q3 Q4 Q1 Q2 Q3 Q4 Q1 Q2 Q3 Q4 Q1 Q2 Q3 Q4 Q1 Q2 Q3 Q4 Q1 Q2


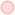

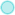
Group Control Intervention Group of points Control Intervention

Scale−up period (omitted from

analysis)

Major Surgeries

90


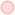

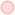
80


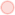


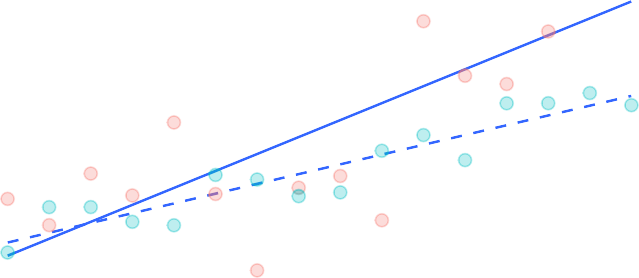

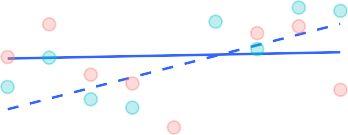
70

60

50


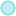


40

30

20

10

0

# Q1 Q2 Q3 Q4 Q1 Q2 Q3 Q4 Q1 Q2 Q3 Q4 Q1 Q2 Q3 Q4 Q1 Q2 Q3 Q4 Q1 Q2 Q3 Q4 Q1 Q2


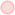

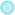
Group Control Intervention Group of points Control Intervention
